# Prolonged Use of a Continuous Peripheral Nerve Block Catheter for Analgesia after Pediatric Foot and Ankle Surgery

**DOI:** 10.1155/2021/8026961

**Published:** 2021-12-21

**Authors:** Jake MacDonald, De-An Zhang

**Affiliations:** ^1^Penn State College of Medicine, Hershey, PA, USA; ^2^Department of Anesthesiology, Shriner Medical Center for Children, Pasadena, CA, USA

## Abstract

Continuous peripheral nerve blocks (CPNB) have a variety of indications and have been shown to be a safe and effective means of minimizing pain postoperatively. Early studies have indicated duration of catheter use greater than 48 hours as a main contributor to infection risk in CPNBs. Recent studies, though, have suggested that the risk of infection does not increase until 4 days after insertion. In the following case report, we recount our experience in using a continuous popliteal-sciatic peripheral nerve block for postoperative pain control in a pediatric patient following calcaneal and first metatarsal osteotomy. The catheter remained in place for 65 hours postoperatively without signs of local inflammation or infection. The prolonged CPNB use resulted in a significant decrease in postoperative opioid use and pain and increase in patient satisfaction when compared to the same procedure done one year prior on the opposite foot.

## 1. Introduction 

Foot and ankle surgery is often associated with postoperative opioid use which has been demonstrated to be associated with several postoperative complications [1]. Continuous peripheral nerve blocks (CPNB) are a safe and effective method in pain management for outpatient foot and ankle surgery [2]. CPNBs have also been shown to reduce the requirement of rescue opioid use following ambulatory orthopedic surgery in comparison to single-shot peripheral nerve blocks (PNB) [3]. Early work has suggested that continuous peripheral nerve block catheters should be removed after 48 hours due to increased risk of infection [4]. Here, we present our experience in postoperative pain control in one patient following a calcaneal osteotomy and first metatarsal osteotomy using a prolonged popliteal-sciatic CPNB. This project was undertaken as a Case Report Project at Shriners Hospitals for Children and, as such, was not formally supervised by an institutional review board. Verbal consent was obtained from the patient's parents prior to publication.

## 2. Case Presentation

Our patient is a 14-year-old male who underwent a calcaneal osteotomy and first metatarsal osteotomy on the left foot 1 year ago and is scheduled for the same procedure now on his right foot. Our patient weighed 58 kg for the first operation. Single-shot adductor canal and popliteal-sciatic nerve blocks, using a 30 mL 0.2% ropivacaine plus 30 mcg perineural dexmedetomidine divided equally between both sites, were performed for postoperative pain control for his first operation. The family reported that the duration of analgesia lasted approximately 24 hours, following which the patient reported severe and prolonged pain requiring usage of 15 tabs of 5 mg oxycodone over a span of approximately 1 week.

At current, our patient's weight has increased to 70 kg. Given his prior poor experience with pain control, we ultimately decided to use a continuous popliteal-sciatic nerve block running 0.2% ropivacaine at 10 mL/h for 65 hours for postoperative analgesia. Oxycodone and diazepam were prescribed for breakthrough pain and muscle spasm in the event of catheter failure or if pain persisted after catheter removal. The popliteal-sciatic catheter was placed and bolused with 15 mL 0.2% ropivacaine through the catheter prior to incision. Tip of the catheter along with local anesthetic spread were visualized under ultrasound. Additionally, a single-shot adductor canal nerve block using 15 mL 0.2% ropivacaine plus 35 mcg perineural dexmedetomidine was performed for cutaneous coverage of the saphenous nerve. The patient experienced no pain in recovery and was discharged home. Follow-up with the parents of the patient occurred daily via telephone calls or text messaging. After the infusion completed 65 hours after insertion, the catheter was removed by the parents without difficulty and discarded. There was no evidence of infection at the catheter site during infusion or after removal of the catheter. Though patient did not have pain, the family did prophylactically give the patient 2 doses of oxycodone and diazepam after the catheter was removed because of the pain experience following the prior surgery. One week after surgery, the patient still did not experience any pain and had not used any additional oxycodone or diazepam.

## 3. Discussion

CPNBs allow for increased flexibility in duration and intensity of blocks in comparison to single-shot PNBs postoperatively. Early studies have shown that the risk of infection increases following 48 hours with an indwelling peripheral nerve block catheter [4]. A more recent study has shown that the likelihood of infection-free peripheral catheter decreases from 99% at day 4 to 96% at day 7, to 73% at day 15 [5]. This study concluded that catheters should be removed after 4 days or as soon as practical to mitigate the chances of infection. In addition to catheter duration greater than 48 hours, previous studies have also indicated intensive care unit stay, trauma patients, femoral or axillary site of insertion, anesthetic solution contamination, absence of antibiotic prophylaxis, and frequent dressing changes as potential risk factors for infection [4, 5]. Mindfulness of these recognized risk factors when leaving a catheter inserted longer than 48 hours is necessary for minimization of infection risk. In order to decrease the overall risk for CPNB catheter infection, insertion sites should be inspected daily for signs of mild infection such as redness, swelling, or localized pain. Additionally, the risk for serious infection can be reduced by removal of the catheter at initial recognition of local inflammation.

**Figure 1:**
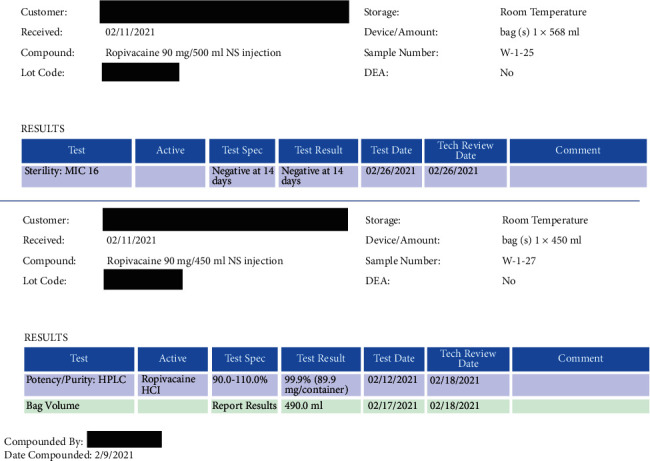
Results of anesthetic solution testing by outside laboratory. Top portion reports no microorganism growth at 14 days. Bottom portion reports 99.9% potency of solution after 4 days.

In our ambulatory surgery center, catheters are placed in the operating room using sterile technique. Insertion sites are then covered and reinforced using sterile, clear, occlusive dressings. We avoid placement of femoral and axillary catheters due to increased risk of infection. Single-dose antibiotic prophylaxis for surgical site infection is used in all cases involving catheters. Our catheters, Pajunk E-Cath, are rated by the manufacturer for 168 hours of indwelling time (PAJUNK GmbH Medizintechnologie, Geisingen, Germany). Our anesthetic solution is sterilely prepared by in-house pharmacy and was tested by an outside laboratory (Dynalabs LLC, St. Louis, MO) showing 99.9% potency up to 4 days and sterility up to 14 days (Figure 1). Our practice has extended the use of indwelling catheters from 48 hours up to 96 hours. Over the past year, we have not had reports of difficult to remove catheters or concerns for infection. As materials vary by institution, it is important for anesthesia providers to review the recommended indwelling ratings of their catheters, sterility of their local anesthetic solutions, and insertion technique prior to making changes to practice and policy.

Our experience of placing peripheral catheters for regional anesthesia supports the body of evidence that CPNBs are safe and effective beyond 48 hours and up to at least 4 days. We would also suggest that, if given an appropriate clinical scenario with the proper precautions taken, indwelling catheters could be used safely up to one week for postoperative pain control.

## Data Availability

The data that support the conclusions of this case report are restricted by HIPPA in order to protect patient privacy and are available from the corresponding author upon request for researchers who meet the criteria for access to confidential data.
